# Fabrication and Microassembly of a mm-Sized Floating Probe for a Distributed Wireless Neural Interface

**DOI:** 10.3390/mi7090154

**Published:** 2016-09-01

**Authors:** Pyungwoo Yeon, S. Abdollah Mirbozorgi, Bruce Ash, Helmut Eckhardt, Maysam Ghovanloo

**Affiliations:** 1 GT-Bionics lab, School of Electrical and Computer Engineering, Georgia Institute of Technology, Atlanta, GA 30308, USA; pyeon6@gatech.edu (P.Y.); smirbozorgi3@gatech.edu (S.A.M.); 2 Premitec. Inc., Raleigh, NC 27606, USA; ash@premitec.com (B.A.); eckhardt@premitec.com (H.E.)

**Keywords:** free-floating wireless implantable neural recording (FF-WINeR), neural recording, wireless power transmission, mechanical biocompatibility, distributed probes, brain mapping

## Abstract

A new class of wireless neural interfaces is under development in the form of tens to hundreds of mm-sized untethered implants, distributed across the target brain region(s). Unlike traditional interfaces that are tethered to a centralized control unit and suffer from micromotions that may damage the surrounding neural tissue, the new free-floating wireless implantable neural recording (FF-WINeR) probes will be stand-alone, directly communicating with an external interrogator. Towards development of the FF-WINeR, in this paper we describe the micromachining, microassembly, and hermetic packaging of 1-mm^3^ passive probes, each of which consists of a thinned micromachined silicon die with a centered Ø(diameter) 130 μm through-hole, an Ø81 μm sharpened tungsten electrode, a 7-turn gold wire-wound coil wrapped around the die, two 0201 surface mount capacitors on the die, and parylene-C/Polydimethylsiloxane (PDMS) coating. The fabricated passive probe is tested under a 3-coil inductive link to evaluate power transfer efficiency (PTE) and power delivered to a load (PDL) for feasibility assessment. The minimum PTE/PDL at 137 MHz were 0.76%/240 μW and 0.6%/191 μW in the air and lamb head medium, respectively, with coil separation of 2.8 cm and 9 kΩ receiver (Rx) loading. Six hermetically sealed probes went through wireless hermeticity testing, using a 2-coil inductive link under accelerated lifetime testing condition of 85 °C, 1 atm, and 100%RH. The mean-time-to-failure (MTTF) of the probes at 37 °C is extrapolated to be 28.7 years, which is over their lifetime.

## 1. Introduction

As neural interfaces strive to more effectively interact with the brain, neural recording and modulation of the future will need the ability to simultaneously interface with multiple sites distributed across large areas of the brain [1,2]. These simultaneous and broad-area neural signal acquisitions are expected to realize brain machine interfaces (BMI) that can restore motor ability to people with severe paralysis [3] and cognitive functions, which are believed to engage different areas of the brain. They can also be used as advanced neuroscience research tools to study the underlying mechanisms of perception, cognition, memory, actions, and emotions, as well as the biomarkers and root causes of many brain disorders, such as Parkinson’s, Alzheimer, and epilepsy, which may affect the interactions among large neuronal populations in widespread networks [4,5,6]. Even though both localized and distributed neural interfacing with large neural ensembles have been demonstrated, current neural interfaces that are clinically viable, fall short of achieving this goal because of their limited coverage of the brain area [6,7,8,9,10].

A major barrier on the path to clinical viability of the intracortical BMIs is their failure in neural signal acquisition over extended periods in the order of the patients’ lifetime [11]. Multiple failure mechanisms have been identified, such as mechanical damage to electrodes and the surrounding tissue, electrical contact corrosion, degradation of the insular coating, and several biological failures, including bleeding, cell death, infection, meningitis, gliosis, and encapsulation, resulting in neuro-inflammatory response [11,12]. Among these failure modes, biological failures are arguably the most difficult ones to deal with, and the ones that we are focusing on. It is found that the micromotions between the tethered electrodes with rigid body and the brain, as a result of brain movements within its surrounding cerebrospinal fluid (CSF), can damage the blood-brain barrier (BBB) and cause inflammation and scar formation around the electrodes, resulting in Signal to Noise Ratio (SNR) degradation, cell death, and shortened lifetime [13,14]. It has been shown that compliant electrode substrates, particularly those with hydrogel or conductive polymer coatings, can reduce the mechanical mismatch with brain tissue, and minimize the motion-induced injury of the tissue due to their flexibility [15,16,17,18]. It has also been demonstrated that anchored probes with tethering on the brain surface to a skull connector or a large implant can exacerbate inflammation and scar formation [19,20].

To replace the existing centralized, and thus large and anchored, neural interfaces, a new class of stand-alone BMIs are under development based on the idea that sufficiently small untethered implants with smooth surface and rounded corners, which are free-floating with the brain, can reduce these effects, improve biocompatibility, and extend the electrodes effective lifetime [21,22]. Tens to thousands of distributed free-floating probes enable massive data acquisition from large neuronal populations across the entire brain. It has been suggested that ultrasonic waves are the most suitable method to deliver power to and acquire data from these small probes [23]. Ultrasound, however, experiences considerable attenuation in bone, imposing heavy constraint on the design, size, efficiency, and complexity of such a BMI, which requires a 2-stage electromagnetic (EM) and ultrasonic wireless power delivery from outside body to intracranial space across the skull and from the surface of brain to implanted probes across the neural tissue, respectively [23]. To address the aforementioned problems, we introduce the concept of a distributed free-floating wireless implantable neural recording (FF-WINeR) system, which is powered via EM in near-field, using magnetic resonance.

**Figure 1:**
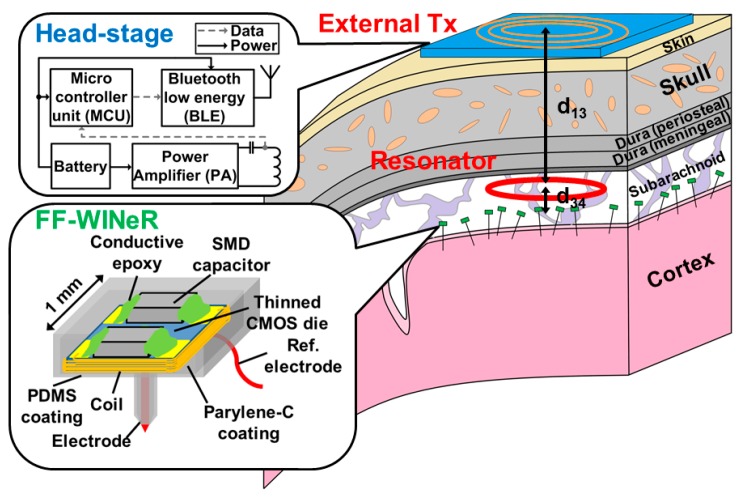
A conceptual diagram of the free-floating wireless implantable neural recording (FF-WINeR) system. Tens to Hundreds of FF-WINeRs are free-floating over the cortex area of interest, like little pushpins, wirelessly powered by the external power amplifier (PA) in the headstage through a 3-coil inductive link. The headstage also collects neural recording data from FF-WINeRs and relays them to a server for post-processing through a Bluetooth Low Energy (BLE) link.

Figure 1 shows a conceptual diagram of the FF-WINeR system, which consists of tens to hundreds of EM-powered mm-sized free-floating probes with a small number of recording electrodes in the form of small pushpins, distributed across the areas of interest on the brain. In the current proof-of-concept prototype, each FF-WINeR probe includes one to four sharpened penetrating Tungsten microwire electrodes for neural recording, a soft-wire non-penetrating reference electrode, a thinned micromachined silicon die that serves both as the probe substrate for mechanical support and houses the application-specific integrated circuit (ASIC), two surface mount device (SMD) capacitors mounted on the silicon die, a bonding-wire coil wound around the die, and hermetic packaging including parylene-C and polydimethylsiloxane (PDMS) coatings. Neural signals are recorded by the electrode(s) and conditioned by an application specific integrated circuit (ASIC) on the CMOS die. In the final version of the FF-WINeR, the ASIC will be powered through a 3-coil inductive link formed between the external coil above the scalp (*L*_1_), the resonator coil underneath dura (*L*_3_), and the receiver coil around the ASIC (*L*_4_) [24]. The same coil, *L*_4_, will be used as an antenna to transmit raw neural data across a short distance to a headstage on top of the head. The headstage relays the neural data to a common server for post-processing through a microcontroller unit (MCU) with built-in Bluetooth Low Energy (BLE). The battery-powered headstage also includes an efficient power amplifier (PA) that supplies power to tens to hundreds of FF-WINeRs, distributed over wide cortical areas of interest, through the 3-coil inductive link.

The fabrication and assembly of the FF-WINeR probe are key aspects of the design that must be addressed at the early stages of development because they directly affect the design decisions made for the other components, such as area and power available for the active circuitry. Exploring the assembly and packaging of the mm-sized passive floating probe, which are similar to the final probes in every aspect, except for the ASIC, is necessary to discover the recording failure mechanisms due to hermetic sealing, manual handling, surgical procedure, or the adverse effect of foreign body reaction and scaring in the brain tissue. Even though there have been many studies concerning brain tissue response to insertion and presence of implantable neural interfacing devices, these were primarily constrained to traditional tethered electrodes [13,25,26,27]. A study of cerebral astrocyte response with untethered electrode in [28] was an early step towards discovering accurate mechanisms of tissue reactivity toward antibodies and brain tissue response with respect to the actual size of the untethered implantable medical devices (IMDs).

In this paper, we focus on microfabrication, assembly, wireless powering, and wireless hermeticity testing of the mm-sized FF-WINeR probes. Hermetically sealed passive probes can be utilized to explore their longevity and robustness in-vitro for future experiments toward the actual FF-WINeR system implementation and in-vivo testing. Section 2 presents considerations for power delivery to mm-sized floating devices. Section 3 describes the microfabrication/assembly methods and hermeticity testing, followed by conclusions in Section 4.

## 2. Power Delivery Considerations in Arbitrarily Distributed mm-Sized FF-WINeR Probes

A comparative study shows that inductive power transfer has the highest power density among several power sources for mm-sized implantable medical devices (IMDs) [29]. To energize mm-sized IMDs, power can be delivered through a 2-, 3-, or 4-coil inductive link [30]. Among these, the 2-coil inductive link is the one traditionally used to power cm-sized IMDs. To the best of our knowledge, the maximum reported power transfer efficiency (PTE) to 1 mm^2^ IMDs via 2-coil inductive link with 1 cm transmitter-to-receiver (Tx-Rx) coil separation is in the order of 1% [31,32]. The 2-coil inductive link is not suitable for supplying power to multiple IMDs at large coupling distances. To deliver sufficient power to multiple arbitrarily distributed IMDs, the 3-coil inductive link is a better choice owing to the effects of magnetic resonance, through the high quality-factor (*Q*) resonator (*L*_3_, *C*_3_), which offers considerably wider coverage and higher PTE [30].

The minimum required power delivered to a load (PDL), based on power consumption of a recent state-of-art neural recording ASIC is ~80 μW per channel: 2.6 μW for analog front-end (AFE) [21], 2.4 μW for transmitter (Tx), 10.6 μW for clock recovery, and 64.1 μW for power management [33]. In this section, we explore the 3-coil inductive link design parameters for a square-shaped receiver (Rx) coil with 1-mm side length.

### 2.1. Design Parameters for 3-Coil Inductive Link Powering a Miniaturized Rx Coil

**Figure 2:**
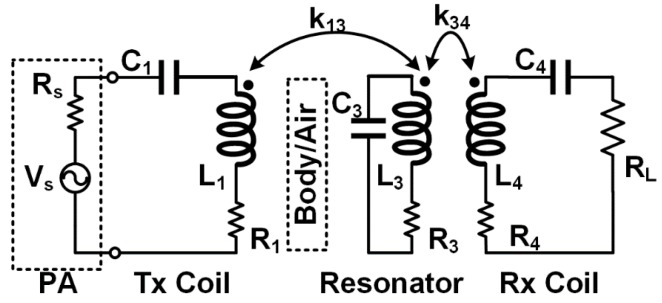
Simplified circuit diagram of 3-coil inductive link for powering the FF-WINeR probes.

Figure 2 shows a simplified schematic diagram of a 3-coil inductive link. *V*_s_ and *R*_s_ represent the PA and its output resistance, respectively. The PA drives the Tx coil (*L*_1_) that is coupled to the resonator coil (*L*_3_) with coupling coefficient *k*_13_, which is in turn encompassing and coupled to one or more Rx coils (*L*_4_) with coupling coefficient *k*_34_. *R*_1_, *R*_3_, and *R*_4_ represent parasitic resistances of *L*_1_, *L*_3_, and *L*_4_, respectively, and R_L_ represents the load resistance. Using tuning capacitors, *C*_1_, *C*_3_, and *C*_4_, all coils are tuned at ω_0_, the frequency of the sinusoidal carrier signal that is generated by *V*_S_ at the PA output.

The PTE from *L*_1_ to the resonator coil, *L*_3_, can be determined as [30],

(1) $$\eta_{13}=\frac{k_{13}^{2}Q_{1}Q_{3}}{1+k_{13}^{2}Q_{1}Q_{3}+k_{34}^{2}Q_{3}Q_{4L}}$$

where *Q*_1_ = ω_0_*L*_1_/*R*_1_, *Q*_3_ = ω_0_*L*_3_/*R*_3_, and *Q*_4L_ = *Q*_4_*Q*_L_/(*Q*_4_ + *Q*_L_), in which *Q*_4_ = ω_0_*L*_4_/*R*_4_ and *Q*_L_ = *R*_L_/ω_0_*L*_4_. *Q*_1,_ *Q*_3,_ and *Q*_4_ are the quality factors of the coils and *Q*_L_ is the load quality factor. PTE between *L*_3_ and the Rx coil(s), *L*_4_, can be approximated as [32],

(2) $$\eta_{34}\approx k_{34}^{2}Q_{3}Q_{4L}\frac{R_{L}}{R_{L}+R_{4}}$$

To maximize η_total_ = η_13_ × η_34_, *L*_3_-*L*_4_ link should be optimized first, followed by *L*_1_-*L*_3_ link. More specifically, we optimize *Q*_4L_ with pre-determined geometry of *L*_4_, which is defined by the size of ASIC in this application. *k*_34_ is highly dependent on the geometries of *L*_3_ and *L*_4_, and less so on their number of turns since both their mutual- and self-inductances of *L*_3_ and *L*_4_ increase with their number of turns [34]. The distances between *L*_1_-*L*_3_ and *L*_3_-*L*_4_, represented by *d*_13_ and *d*_34_, respectively, are the other key parameters affecting PTE and PDL, which nominal values (*d*_13_ = 28 mm, *d*_34_ = 0 mm) are selected based on the human head and brain anatomy, as shown in Figure 1.

### 2.2. Bonding-Wire Wound Coil Design for FF-WINeR Probes and 3-Coil Inductive Link Design

The size of Rx coil is often limited by constraints of the application [35,36]. For distributed neural interfaces, the Rx coil size is restricted to a few millimeters. The smaller implant size reduces scar formation and inflammation, however, it also lessens PTE/PDL of the inductive link. As a compromize between PTE, specific absorption rate (SAR)-constrained PDL, and potential tissue damage, the side length of FF-WINeR Rx coil was chosen to be be 1 mm [32]. The target load resistance is chosen 9 kΩ, which is the equivalent of delivering 111 μW to the load when the voltage across the load is 1 V_rms_. Under these constraints, the paramters in the Rx coil that can be optimized are the coil pitch, wire diameter, and number of turns. With the choice of an insulated bonding-wire (Microbonds Inc., Markham, ON, Canada), wire diameter is fixed at 25 μm. The coil pitch is somewhat difficult to control when the coil is wound using a manual a wire-bonding machine. Finally, the optimal number of turns is chosen to maximize *Q*_4L_ using the High Frequency Structural Simulator (HFSS, ANSYS Inc., Canonsburg, PA, USA).

**Figure 3:**
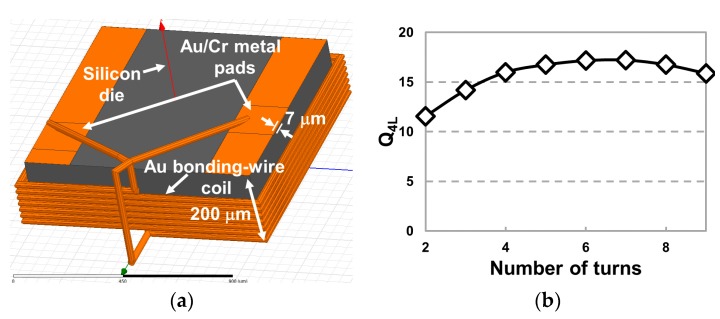
(**a**) High Frequency Structural Simulator (HFSS) simulation model of Rx bonding-wire coil with 25 μm gold core diameter, wound around a 1 mm × 1 mm × 0.2 mm piece of silicon that provides mechanical support for the coil and substrate for the application-specific integrated circuit (ASIC). Two ends of the wire are ultrasonically bonded to a pair of gold metal pads with 7 μm thickness. The center-to-center coil pitch in this model is 30 μm. (**b**) *Q*_4L_ vs. *L*_4_ number of turns at 135 MHz and *R*_L_ = 9 kΩ. Other design parameters are shown in Figure 3a.

The HFSS simulation model for *L*_4_ is shown in Figure 3a. Gold bonding-wire with Ø25 μm core is wrapped around a 1 mm × 1 mm × 0.2 mm silicon (Si) die and two ends of the wire are ultrasonically bonded to 7 μm thick gold pads on the Si die. The coil pitch is set at 30 μm for this simulation even though it is not easily adjustable during manual fabrication. At this stage of the research, when regulatory compliance is not yet considered, the inductive link optimization often involves selection of the optimal carrier frequency by sweeping the frequency while measuring the PTE. We chose 135 MHz within the optimal operating frequency range, 120–200 MHz in [31,32] and considering the trade-off between the link PTE and the rectifier power conversion efficiency (PCE). Figure 3b illustrates *Q*_4L_ vs. number of turns of *L*_4_ at 135 MHz, which varies from 11.5 to 17.2, indicating that the optimal number of turns is 7.

There is a trade-off between the number of FF-WINeRs that fit inside each resonator, set by the diameter of the resonator and η_34_. Here we have considered the outer resonator diamer, *D*_o3_ = 3.6 cm, to provide enough room for about 100 FF-WINeRs inside. The outer diameter of the Tx coil, *D*_o1_, is derived considering the distance between the Tx and resonator coils, *d*_13_ = 2.8 cm [37],

(3) $$D_{o1}=\sqrt{D_{o3}^{2}+4\times d_{13}^{2}}$$

### 2.3. Wireless Power Transmission (WPT) to FF-WINeR Probes

**Figure 4:**
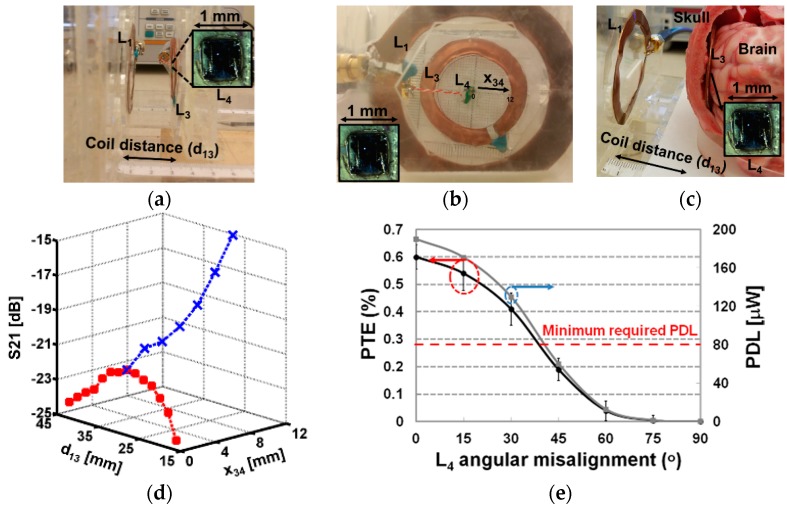
(**a**) The experimental setup to measure S21 from the transmitter (Tx) to receiver (Rx) coil at different coil separations, *d*_13_, in air; (**b**) The experimental setup to measure S21 depending on the horizontal misalignment of the Rx coil from the center of the resonator, *x*_34_, in air; (**c**) The experimental setup using a lamb head to emulate the neural tissue environment; (**d**) The measured S21 of the 3-coil inductive link at 137 MHz in air; (**e**) Power transfer efficiency (PTE) and power delivered to a load (PDL) of the 3-coil inductive link in the lamb head tissue medium versus angular misalignment of *L*_4_ (*x*_34_ = 0 mm, *d*_13_ = 28 mm). PTE measurements were repeated 6 times and error bars show 95% confidence interval.

The 3-coil inductive link is implemented based on the above design considerations to explore the achievable PTE and PDL versus the coil distance between *L*_1_ and *L*_3_ (*d*_13_), and *L*_4_ horizontal misalignment from the center of *L*_3_ (*x*_34_), as shown in Figure 4a,b. The electrical and geometrical characteristics of the coils are summarized in Table 1.

The S-parameters are measured from *L*_1_ to *L*_4_ in the presence of *L*_3_ on the same plane with *L*_4_ using a ZVB4 vector network analyzer (Rohde & Schwarz, Munich, Germany). The twisted red-white feeding line to *L*_4_ in Figure 4b introduces parasitic coupling, inductance, and resistance to those of *L*_4_, and can diminish the measurement accuracy. Therefore, we have adopted a calibration method, known as de-embedding, which is described in [32]. The de-embedded S21 of the 3-coil inductive link at 137 MHz versus *x*_34_ and *d*_13_ in the air is depicted in Figure 4d. If *x*_34_ = 0 mm, the min and max S21 are −24.45 and −21.2 dB when *d*_13_ = 42 mm and 28 mm, respectively. At *d*_13_ = 28 mm, the nominal Tx-resonator coils’ distance that maximizes S21, the min and max of S21 are −21.2 dB and −15.1 dB when *x*_34_ = 0 mm and 12 mm, respectively. Similarly, the minimum S21 is measured −22.26 dB in a lamb head medium, as shown in Figure 4c, when *x*_34_ = 0 mm and *d*_13_ = 28 mm. Based on the measured S21 and S11, the PTE can be derived as [38],

(4) $$\mathrm{PTE}=100\times \frac{\left|S21{|}^{2}\times (1+\frac{Z_{0}}{R_{L}}\right)}{1-|S11{|}^{2}},$$

where *Z*_0_ is the characteristic impedance, 50 Ω. The achieved minimum PTE (with no rotation) in the air and lamb head medium using Equation (4) are 0.76% and 0.6%, respectively. Since the source power of the vector network analyzer is fixed at 15 dBm, the achieved minimum PDL in the air and neural tissue medium can be calculated as 240 and 191 μW, respectively. This level of PDL is sufficient to operate the state-of-art neural recording ASICs with small number of channels [33].

The brain surface is curved and filled with gyri and sulci, which make it unlikely for the small mm-sized *L*_4_ to be perfectly aligned with *L*_3_. This is expected to reduce the achievable minimum PTE and PDL of the 3-coil inductive link due to angular misalignment between *L*_3_ and *L*_4_. We measured the PTE and calculated PDL of the proposed link while rotating *L*_4_ from 0° to 90° in the lamb head tissue medium. The measurement was repeated 6 times for better accuracy because of difficulty in setting the angular rotation of *L*_4_ in the lamb head. Figure 4e shows PTE and PDL versus *L*_4_ angular misalignment at *x*_34_ = 0 mm and *d*_13_ = 28 mm. With 15° *L*_4_ angular misalignment, PTE and PDL decease to 0.54% and 171 μW, respectively, with ~10% measurement error. They drop further down to 0.2% and 60 μW, respectively, with 45° misalignment. Therefore, for state-of-art operating power of 80 μW in [21,33], the maximum tolerable angular misalignment in this WPT paradigm is ~40°.

For chronic implantation, the thermal elevation in the brain tissue surrounding the proposed 3-coil inductive link should be considered, even though a detailed modeling and measurement of thermal effects are out of the scope of this manuscript. Since PDL to *L*_4_ is only 191 µW, its temperature elevation is negligible. On the other hand, circuit simulation using the fabricated coil parameters in Table 1 indicate 31 mW power dissipation in *L*_3_ under abovementioned operating conditions. Considering the model and measurements presented in [39], if this amount of power was dissipated in a traditional brain implant with 0.55 cm^2^ surface area, is will result in ~1.5 °C. However, the surface area of *L*_3_ is 15 times larger than the implant presented in [39], and it is likely to have better thermal conductivity than silicon. Therefore, we roughly estimate a temperature rise in the order of 0.1 °C.

## 3. FF-WINeR Passive Probe Fabrication and Hermeticity Testing

Fabrication of the FF-WINeR probes requires a novel process flow due to the extremely small size of the device. The process includes micromachining, microassembly, and hermetic packaging. Fabricated probes should undergo a hermeticity testing to estimate their lifespan under harsh operating conditions inside the body.

### 3.1. Silicon Die Fabrication Process

**Figure 5:**
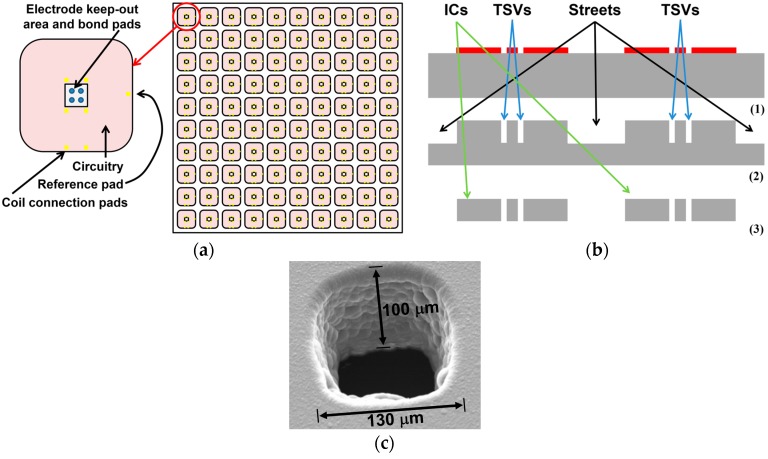
(**a**) Top view of the DRIE mask and additional features of the Si die; (**b**) Cross-sectional view of the Si die fabrication steps; (**c**) Scanning electron microscope (SEM) photo for a 130 µm through-silicon-via (TSV) in the middle of each Si die.

For passive Si die fabrication, ultraviolet (UV) photolithography is used to define an array of 1 mm^2^ dice on a blank Si wafer, based on previously developed processes described in [40,41,42,43]. As shown in Figure 5a,b, the patterning defines through-silicon vias (TSVs) on the same mask, which eventually accept the microwire electrodes, while streets are used to segregate the dice from one another. The die is designed with rounded corners for smooth implant surface and better coating and biocompatibility. The exposed silicon streets and TSVs are etched in a deep reactive ion etcher (DRIE) using a modified Bosch process to depth of 100 μm—the desired Si die thickness in the FF-WINeR probes, as shown in Figure 5b(2). This wafer is flipped upside-down, and adhered to a sacrificial wafer using Revalpha high temperature thermal release tape. The backside of the silicon is then etched away as shown in Figure 5b(3), exposing the TSVs and streets etched previously, thus opening the TSVs and segregating the individual dice. Figure 5c is a scanning electron microscope (SEM) photo of the TSV. The FF-WINeR ASIC will be designed by leaving a 270 μm × 200 μm blank area in the center of the die to accommodate placement and electrical connections of microwire electrodes. The original 11 mm × 11 mm ASIC will be fabricated at a foundry in a CMOS-compatible process with a 10 × 10 array of FF-WINeRs, as shown in Figure 5a, such that each diced die produces as many as 100 FF-WINeR ASICs after post-processed, as described above. The only difference would be the use of an additional sacrificial wafer throughout the process to facilitate photolithography and micromachining of diced ASICs.

### 3.2. mm-Sized FF-WINeR Probes Microassembly

**Figure 6:**
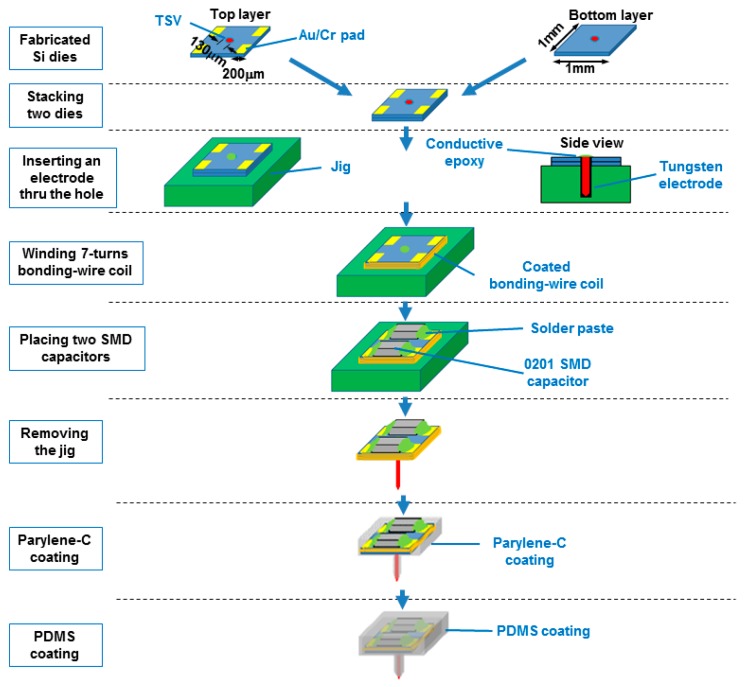
FF-WINeR passive device fabrication procedure. Two processed Si dies (1 mm × 1 mm × 100 μm) are stacked together and a tungsten electrode with 81 μm diameter is inserted through a hole in the middle of the stacked Si dies. A coil is wound around the Si dies with coated bonding-wire. The device is coated with PDMS and parylene-C.

The procedure to microassemble the FF-WINeR probes is illustrated in Figure 6. Depending on the complexity of the ASIC, one or two Si dies fabricated through the process described in Section 3.1 are stacked, carefully aligned, and glued or ultrasonically bonded together. The stacked dice are placed in a jig made from a quad-flat no-leads (QFN) package (Quik-Pak, San Diego, CA, USA) with a hole in the middle to allow for insertion of the electrode. The tungsten electrode, Ø81 μm in diameter including 3 μm thick Teflon insulation with a tapered tip processed by electrochemical etching, parylene deposition, and arc exposure (Microprobes, Gaithersburg, MD, USA), is inserted through aligned holes, which the electrode back-end is affixed to the large metal pad ring around the hole using conductive epoxy. In the active version of the probe, this pad is connected to the low noise amplifier block of the ASIC.

**Figure 7:**
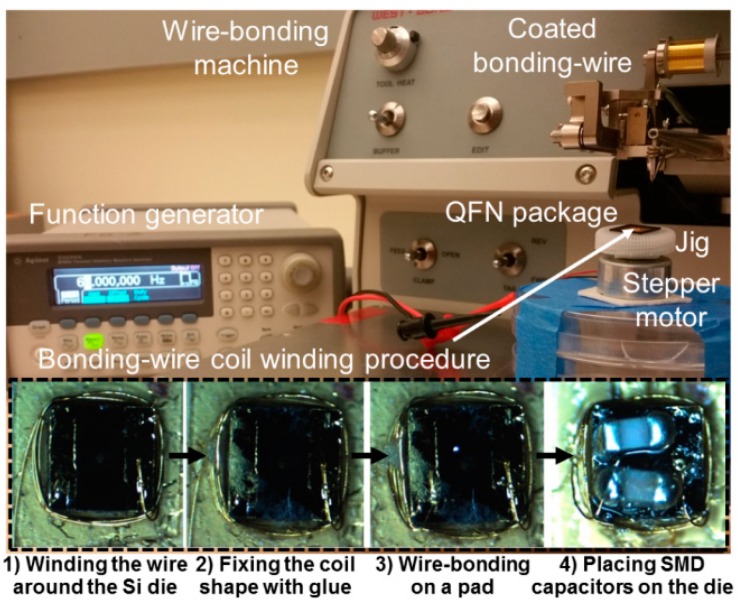
Semiauto wire-bonder for bonding-wire wound coil and steps for winding a coil (in the dashed box). A QFN package is glued on top of the jig and rotating on the stepper motor powered by the Agilent 33250A function generator (Keysight, Santa Rosa, CA, USA). Wire-bonding machine is utilized to wind a coil with a coated bonding-wire around the Si die taped on the QFN package.

Implementing the mm-sized *L*_4_ is one of the key steps in passive probe fabrication. One way is to utilize a fully-automated wire-bonder which bonding tip is programmed to move along a pre-defined trajectory around the core [44]. For prototyping, however, we have developed a simpler setup using a manual wire-bonder (Westbond 7476D, Anaheim, CA, USA) and a stepper motor, shown in Figure 7. The process, which is presented in the insets at the bottom of Figure 7, starts with the stacked Si dies fixated in the QFN package jig. One end of the wire is ultrasonically bonded on its associated metal pad, while the jig is slowly rotated by the stepper motor. While the Si die is rotating, the wedge position is manually adjusted around the probe back-end until the desired number of turns is reached. To maintain mechanical integrity of the coil, small amount of Loctite 4014 instant glue (Henkel Corp., Rocky Hill, CT, USA) is added on four corners of the coil. Then, the other end of the coil wire is ultrasonically bonded to the other metal pad on the die. Finally, the two 0201 SMD capacitors (Murata, Kyoto, Japan) are mounted on the Au pads over the upper Si die with low temperature solder paste before starting the coating steps. One, *C*_4_, is a tuning capacitor to resonate with *L*_4_ at the operating frequency and the other is the load capacitor, *C*_L_, low-pass filtering the output of a voltage doubler for AC-to-DC conversion.

**Figure 8:**
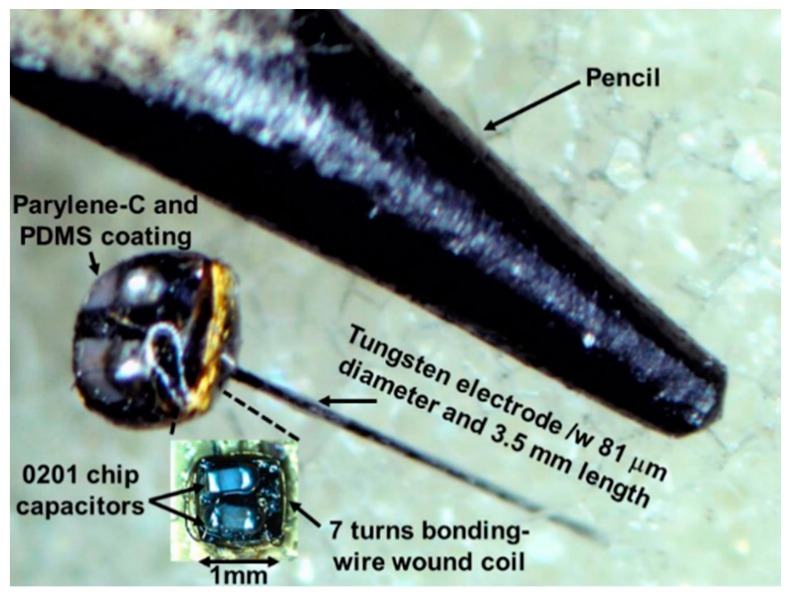
Fabricated passive FF-WINeR prototype. 7 turns boding-wire coil is wrapped around a stack of two 100 µm thick Si dies. A tungsten electrode with 81 μm diameter and 3.5 mm length is inserted through a Ø130 μm TSV in the center of the dice. There is also enough room for two 0201 SMD capacitors on the upper Si die. The entire device is coated with parylene-C and PDMS for hermeticity and biocompatibility.

To hermetically seal the FF-WINeR against the harsh environment in the body and maintain biocompatibility, a 5-μm thick parylene-C coating is applied on the assembled passive probe by vapor deposition (SCS Labcoater 2 PDS 2010, Specialty Coating Systems, Indianapolis, IN, USA). Even though parylene-C is biocompatible, a layer of Sylgard 184 PDMS coating (Dow Corning Corp., Auburn, MI, USA) is added over parylene-C to extend the probe life-span and create a smooth and soft surface. The uncured PDMS mixture has many trapped air bubbles, which should be removed in a dessicator connected to a vacuum pump until the air bubbles are removed. The passive probe is then dipped in bubble-free PDMS, and hung upside down from a hook with the electrode tip inserted in a piece of styrofoam for at least 24 h to cure at the room temperature. Figure 8 shows the completed mm-sized passive FF-WINeR probe prototype next to the tip of a pencil for size comparison.

### 3.3. Hermeticity Testing for the Packaged FF-WINeR Probes

Since biological fluids contain NaCl, KCl, phosphates, carbonates, enzymes, and other proteins, they create a harsh environment for the probe, while the surface of the probe can create a harmful environment for the surrounding cells [45]. In such an environment, water is likely to ingress into the electronic package or toxic material can leak out, both of which are detrimental to the probe and its delicate surrounding tissue. Here, we present a method for wirelessly testing the hermeticity of the FF-WINeR probes’ packaging.

**Figure 9:**
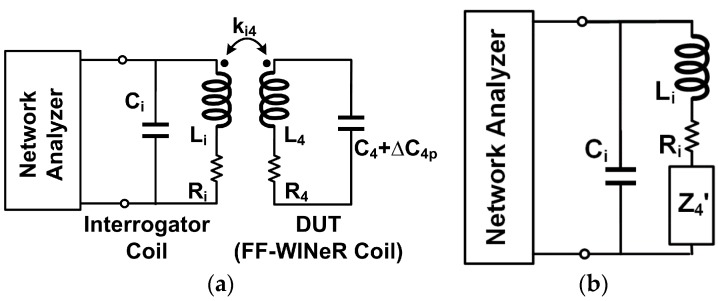
(**a**) The circuit diagram for the wireless hermeticity testing. (**b**) Its equivalent circuit model seen at the interrogator coil side with reflected impedance from the device-under-test (DUT). *k_i_*_4_ is coupling coefficient, *L_i_* is inductance of the interrogator coil, *L_4_* is inductance of the DUT, *C_i_* is parasitic capacitance of *L_i_ R_i_* and *R*_4_ are the parasitic resistance of *L_i_* and *L*_4_, respectively, *Z*^’^_4_ is the device-under-test (DUT) reflected impedance onto the interrogator coil side.

Our hermeticity testing method was inspired by the wireless capacitive sensing presented in [46]. Figure 9a shows the schematic of an interrogator coil, *L_i_*, attached to a network analyzer with the device-under-test (DUT), which is the FF-WINeR Rx *L*_4_*C*_4_ tank coupled by the coupling coefficient, *k*_14_, in this case, placed in proximity. The basic principle is measuring the changes in reflected impedance of *L*_4_ and its tuning capacitance, *C*_4_, plus its parasitic capacitance, Δ*C*_4p_ DUT due to water ingress in the package. *R_i_* and *R*_4_ are the parasitic resistance of *L_i_* and *L*_4_, respectively. The DUT reflected impedance onto the interrogator coil side is depicted in Figure 9b according to the reflected-load-theory (RLT) [47],

(5) Z4′=(ωMi4)2R4+jωL4+1jω(C4+ΔC4p)

where $M_{i4}=k_{i4}\sqrt{L_{i}L_{4}}$ is the mutual inductance between *L_i_* and *L*_4_. Moisture in the package can either nullify *C*_4_ by shorting the SMD capacitor or increase *C*_4p_ by increasing dielectric constant. The relative dielectric of water is 80.1, 29.9 times larger than that of PDMS, which is 2.68 [48]. Water leakage does not affect *L*_4_ since the permeability of water is very close that of vacuum. The electrical characteristics of *L_i_* and *L*_4_ in the current FF-WINeR prototype are summarized in Table 2.

**Figure 10:**
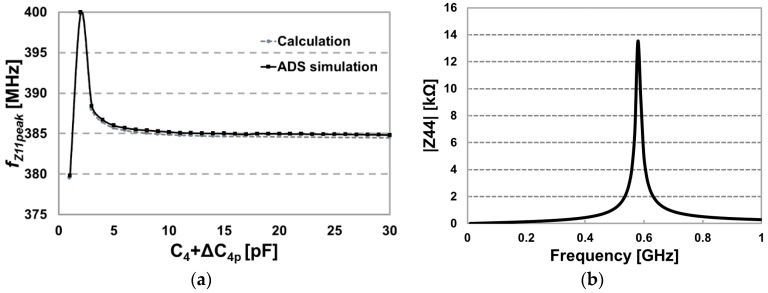
(**a**) The frequency at which Z11 reaches its peak, *f*_Z11peak_, vs. *C*_4_+Δ*C*_4p_ simulated and estimated using Advanced Design System (ADS) and Equation (6), respectively. (**b**) Measured |Z44| frequency response when *L*_4_*C*_4_ is soaked in water. This curve can be used to extract Δ*C*_4p_.

If we calculate Z11, the input impedance seen through *L*_1_, the frequency at which it peaks, *f*_Zpeak_ can be estimated by solving Equation (6) for ω [49],

(6) $$1-{\left(\frac{\omega }{\omega_{i}}\right)}^{2}-{\left(\frac{\omega }{\omega_{4}}\right)}^{2}+\left(1-k_{i4}^{2}\right)\left(\frac{\omega^{4}}{\omega_{i}^{2}\omega_{4}^{2}}\right)=0$$

where ω*_i_* and ω_4_ are the resonant frequencies of *L_i_C_i_* and *L*_4_*C*_4_, respectively. Variations in *f*_Z11peak_, based on solving Equation (6) in Mathematica (Wolfram, Boston, MA, USA), are plotted in Figure 10a against Advanced Design System (ADS, Keysight, Santa Rosa, CA, USA) simulation results. Changes in the dielectric result only in a few pF change in Δ*C*_4p_, shifting *f*_Z11peak_ by less than in a few 100 kHz, which is very small compared to the original *f*_Z11peak_, 384 MHz. On the other hand, the short circuited *C*_4_ can cause considerable frequency shift from 384 MHz down to 379.8 MHz. To estimate *C*_4p_ when *C*_4_ is shorted, the FF-WINeR coil impedance, Z44, was measured when it was soaked in water. Figure 10b shows Z44 variations vs. frequency, using which *C*_4p_ can be estimated ~1 pF when *L*_4_ = 92 nH.

**Figure 11:**
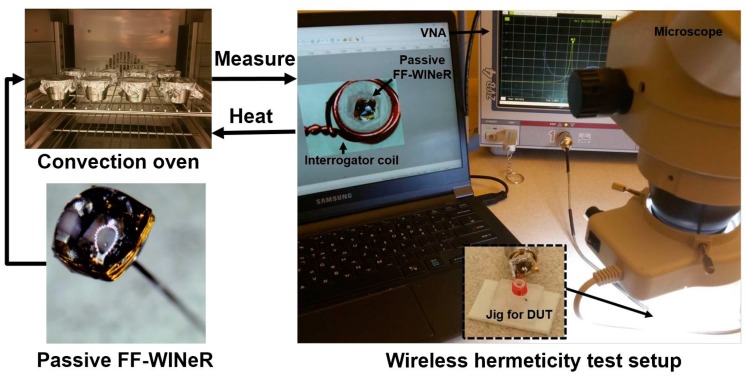
Packaging accelerated lifetime measurement setup: six passive FF-WINeR probes are placed individually in water containers in a convection oven at 85 °C. Probes are periodically taken out of the oven and monitored for changes in Z11 reflected onto interrogator coil, wound around a jig. The frequency at which Z11 peaks, *f*_Z11peak_, is measured by vector network analyzer.

The most accurate method of test the packaging lifetime is placing the DUT in an actual in-vivo operating condition [45]. A simpler, faster, but less accurate method is accelerated lifetime testing, which was conducted on six passive FF-WINeR probes in the convection oven (OF-02G, Jeio Tech, Daejeon, Korea) at 1 atm pressure, 100% RH humidity, and 85 °C temperature. Each packaged probe was submerged in water in a container that was sealed with aluminum foil, as shown Figure 11. Z11 was measured by a vector network analyzer to register the original *f*_Z11peak_ by carefully placing the DUT on a Jig with *L_i_* wound around it under a microscope to make sure the DUT is always placed in the same position. Initially we measure Z11 of each sample every 24 h and gradually increased the period between successive measurements when the critical failure period was passed.

**Figure 12:**
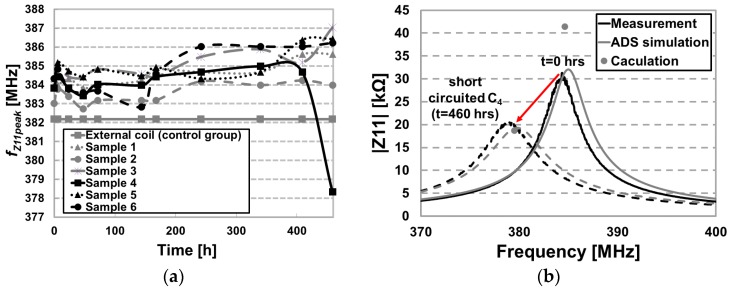
(**a**) Measured *f*_Z11peak_ of six passive FF-WINeR samples plotted over 460 h. *f*_Z11peak_ of the interrogator was measured before and after placing each sample in the jig, shown in Figure 11; (**b**) Frequency shift in the *f*_Z11peak_ by shorting the SMD capacitor of the passive FF-WINeR probe. Black lines: measurement results, Gray lines: ADS simulation results and Gray dots: calculation results of *f*_Z11peak_.

Figure 12a shows the changes in *f*_Z11peak_ for six samples over 19.2 days. The *f*_Z11peak_ of the external coil is monitored every time we measure the reflected Z11 for six samples as a control group so as to reduce the measurement error by equipment inaccuracy. Measurements show that on average *f*_Z11peak_ increased after 242 h. However, this shift seems to have been the result of various measurement error, which can be reduced by designing a more a more robust and reproducible lifetime measurement setup. The *f*_Z11peak_ of sample 4, however, dropped considerably from 384 to 378.3 MHz from after 460 h. Figure 12b compares the average Z11 frequency response of six passive FF-WINeR samples at the initial point and Z11 of sample 4 at 460 h. We contribute this major shift to the water leakage inside the hermetic sealing, shorting the receiver resonance capacitor, C_4_.

In a widely used model for accelerated lifetime testing, the acceleration factor (AF) is defined based on the Arrhenius equation as [50],

(7) $$\mathrm{AF}=\frac{\left(RH^{-n}e^{\frac{\Delta E_{a}}{kT}}\right)\mathrm{normal}}{\left(RH^{-n}e^{\frac{\Delta E_{a}}{kT}}\right)\mathrm{accelerated}}$$

where RH is the relative humidity, *k* is Boltzmann constant, *n* is an empirical constant, ∆*E*_a_ is the activation energy, and *T* is the absolute temperature. The common value of *n* is 3.0 for hermetic sealing tests and ∆*E*_a_ = 0.9 eV for polymer packages. The estimated AF for normal operating condition, i.e., 100% RH and 37 °C, inside the human brain is 91. The lifespan of a device is often estimated using the mean time of failure (MTTF) in years and describes as [51],

(8) $${\mathrm{MTTF}}_{\mathrm{years}}=\frac{1}{\left(\lambda_{\mathrm{hour}}\times 24\times 365\right)}$$

where λ_hour_ is the failure rate per hour that can be defined as,

(9) $$\lambda_{\mathrm{hour}}=\frac{F}{\left(N\times T\times \mathrm{AF}\right)}$$

where *F* and *T* are the number of failures and test hours per device. The MTTF in this case is estimated to be 28.7 years. The small amount of water penetration into the hermetic package may be considered as a hermetic sealing failure. It should be noted that since the passive FF-WINeR has two layers of polymer coating, PDMS over parylene, water ingress into the PDMS layer does not necessarily lead to device failure despite causing a small change in the *f*_Z11peak_. The 5 μm parylene-C coating layer is considerably more resistant to water than the PDMS coating, and maintains functionality of the active circuit even though the *L*_4_*C*_4_ resonance frequency may shift as a result. This shift can be remedied, however, using the auto resonance tuning (ART) method, described in [52].

## 4. Conclusions

We have presented the structure, microfabrication, and assembly steps of a mm-sized implant toward a distributed free-floating wireless implantable neural recording (FF-WINeR) system, which can be used for safer and less invasive neural interfacing with potentially less damage to the neural tissue. The silicon chip that houses the ASIC is micromachined after standard CMOS processing to also acts as a substrate and provide mechanical support for the microwire electrodes and the bonding-wire coil, which is wound around the ASIC to offer higher Q compared to on-chip coils. Wireless power transmission to the FF-WINeR is designed based on 3-coil inductive link, using a high-Q planar implantable resonator in the same plane as the FF-WINeR probes and encompassing them. Preliminary experiments show that the required PTE and PDL for the desired functions in the ASIC are achievable with Tx coil separation of 2.8 cm. A simple method is developed for wireless hermeticity testing of the dual polymer coated FF-WINeR probes based on accelerated lifetime testing technology. We are now developing the ultra-low power and area efficient active circuitry for this probe and further improving the hermeticity testing methodology to achieve more accurate estimates of the FF-WINeR probe lifetime. Future steps also include in-vivo experiments on rat animal model.

## Figures and Tables

**Table 1:** Electrical characteristics and geometric parameters of the 3-coil inductive link.

Parameters (*i* = 1, 3, 4)	Tx Coil (*L*_1_)	Resonator Coil (*L*_3_)	Rx Coil (*L*_4_)
Inductance, *L_i_*	85 nH	38 nH	94 nH
Quality factor, *Q_i_*	400	325	22
Outer diameter, *D*_o*i*_	51 mm	36 mm	1 mm×1 mm
Line width, *w_x_*/thickness, *t_i_*	5 mm/0.2 mm	4 mm/0.2 mm	25 μm (diameter)
Number of turns, *N_i_*	1	1	7

**Table 2:** Electrical characteristics for the interrogator coil and FF-WINeR Rx coil.

Parameters	Interrogator Coil (*L_i_*)	FF-WINeR Rx Coil (*L*_4_)
Inductance	122.9 nH	92 nH
Resistance	0.674 Ω	1.88 Ω
Capacitance	1.41 pF	15 pF
Outer diameter	3 mm	1 mm × 1 mm
Wire diameter	63.5 μm	25 μm
Number of turns	7	7
Coupling coefficient (*k_i_*_4_)	0.11	

## Abbreviations

The following abbreviations are used in this manuscript:

SUA	Single Unit Activity
MEMS	Microelectromechanical systems
Si	Silicon
PTE	Power Transfer Efficiency
PDL	Power Delivered to the Load
SNR	Signal to Noise Ratio
BMI	Brain Machine Interfaces
RF	Radio Frequency
CP	Conducting Polymer
FF-WINeR	Free-floating wireless implantable neural recording system
CMOS	Complimentary metal-oxide-semiconductor
PDMS	Polydimethylsiloxane
ASIC	Application Specific integrated circuit
MCU	Microcontroller unit
BLE	Bluetooth Low Energy
PA	Power amplifier
AFE	Analog Front-End
Tx	Transmitter
Rx	Receiver
SAR	specific absorption rate
HFSS	High frequency structural simulator
PCE	Power Conversion Efficiency
TSV	Through-silicon-vias
DRIE	Deep reactive ion etcher
IC	Integrated Circuits
DUT	Device under test
AF	Acceleration factor
RH	Relative humidity
MTTF	Mean time to failure
